# Analysis of impacts of exogenous pollutant bisphenol-A penetration on soybeans roots and their biological growth†

**DOI:** 10.1039/d2ra08090g

**Published:** 2023-03-28

**Authors:** Eujung Kim, Minjung Song, Adam Gopal Ramu, Dongjin Choi

**Affiliations:** a Department of Materials Science and Engineering, Hongik University 2639-Sejong-ro, Jochiwon-eup Sejong-city 30016 Republic of Korea djchoi@hongik.ac.kr

## Abstract

Bisphenol A (BPA) is a common chemical used in plastic production. BPA, which has the potential to be poisonous to plants, has lately emerged as a serious environmental concern owing to its extensive usage and release patterns. Prior study has only looked at how BPA affects plants up to a certain stage in their growth. The precise mechanism of toxicity, penetration of BPA, and damage to internal root tissues remains unknown. Therefore, the goal of this study was to examine the hypothesized mechanism for BPA-induced root cells by studying the effects of bisphenol A (BPA) on the ultrastructure and function of root tip cells of soybean plants. We looked at plant changes in root cell tissues after BPA exposure. Further, the biological characteristics that responded to BPA stress were investigated, and the accumulation of BPA in the root, stem, and leaf of the soybean plant was systematically investigated by using FTIR and SEM analysis. The uptake of BPA is a key internal factor that contributes to changes in biological characteristics. Our findings provide insight into how BPA could alter plant root growth, which might contribute new knowledge toward a better scientific appraisal of the possible dangers of BPA exposure for plants.

## Abstract 2

Bisphenol A (BPA), a synthetic chemical used in polycarbonate plastic and epoxy resin production, is an endocrine disruptor. It interferes with hormonal functioning to cause adverse effects on reproduction, growth, and development in aquatic and terrestrial organisms.

## Graphical abstract

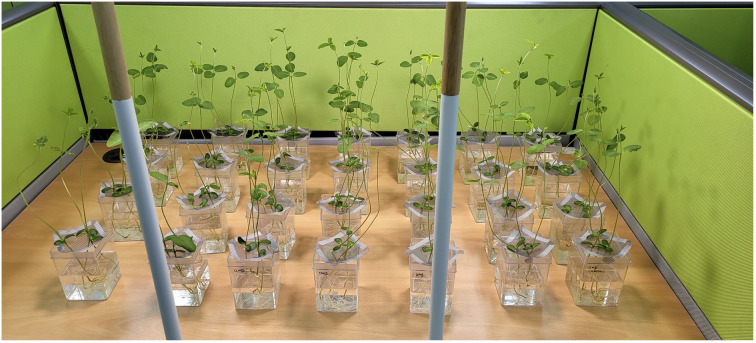

## 1. Introduction

Bisphenol A [2,2-bis(4-hydroxyphenyl)propane; BPA], a common endocrine disruptor, has been widely utilized in the manufacturing of epoxy resin, polycarbonate, and other polymer materials.^1–7^ Because of its widespread production and use, BPA has made its way into the environment. Research has found that the concentration of bisphenol A (BPA) in soil ranges from 2 to 140 g kg^−1^ of dry soil in Europe, the United States, and Korea. BPA has also been detected in the atmosphere of several major cities and in the sediments of rivers all across the globe.^8–11^ Furthermore, the BPA concentrations in the effluent and sludge generated by wastewater treatment plants range from 3 ng L^−1^ to 316 ng L^−1^ and from 0.42 ng g^−1^ dry weight of sludge to 25 600 ng g^−1^ dry weight of sludge, respectively.^12–16^ Finally, 17.2 mg L^−1^ of BPA was discovered in leachate from hazardous waste in Japan.^17^ BPA has been associated with serious reproductive, developmental, neurological, and immunological problems in humans and animals, and toxicity tests in animals have confirmed that it possesses estrogenic-like endocrine-disrupting effects.^18–20^ Because of its widespread presence and presumably increasing levels in the environment, BPA has been labelled a pollutant of rising concern, posing risks to exposed living species (humans, animals, and plants).^21–25^ Many recent studies have examined the outcomes of BPA exposure in both animals and humans. Plant toxicity from this pollutant is significantly less well understood. Plants can provide a sensitive reflection of environmental changes, such as the effects of pollution, because of their intimate association with factors such as climate, temperature, light, and stress. Similarly, activated sewage sludge biosolids are often used to enhance agricultural soils; these biosolids may contain bisphenol A (BPA), which means that plants grown in that soil may be exposed to BPA through soil organisms and potentially dangerous landfill leachates.^26^ Therefore, plants are a good subject for studying the ecological and environmental risks posed by BPA. According to prior research, BPA is a relatively hydrophobic chemical that tends to collect in high-fat plant roots and hence can rarely travel inside plants.^27–30^ Thus, the most prevalent syndrome of BPA phytotoxicity is the suppression of root growth. Several investigations on the phytotoxic effects of BPA have demonstrated that exposure to BPA over a particular dose (>10 mg L^−1^) can cause inhibition of plant root growth in a dose-dependent way, manifested by shortening of root length and fresh/dry weight, sparse lateral roots, and the production of gelatinous substances and blackened necrosis in local roots. Additional research revealed that BPA exposure can interfere with a number of physiological processes in plant roots, including the absorption of nitrogen nutrients and mineral elements, the secretion of endogenous hormones, the formation of reactive oxygen species (ROS), and the operation of antioxidant defence systems.^31–34^

The United States Environmental Protection Agency (2012) suggests using soybean (Glycine max L.) to evaluate pollutant effects in plant species because to soybean's widespread importance as a cash and food crop. For this reason, soybean seedlings were employed as the experimental subject. Soybean root growth after exposure to bisphenol A (BPA) was studied, as were BPA's effects on the ultrastructure and function of mitochondria in root tip cells of soybean, in an effort to shed light on the anticipated mechanism of BPA-induced root cell mitochondrial malfunction. We investigated how plant biological characteristics responded to BPA stress, identified sensitive biological characters, and preliminary examined the internal factors that contribute to changes in biological characteristics by examining plant changes in ultra-structure in root cell tissues after BPA exposure. Our findings may contribute new knowledge toward a better scientific assessment of the possible dangers of BPA exposure to plants by shedding light on how BPA can impact plant root growth.

## 2. Experimental

### 2.1 Chemical and reagents

Bisphenol A (>99.9%) was purchased from Dae-Jung chemical, Korea. The obtained BPA powder was grained by ball-milling process for 2 days. Hoagland and Arnon nutrient solution was obtained from Korea.

### 2.2 Preparation of BPA solution

BPA powder was grained by the ball-milling process for 2 days. Various concentrations of BPA solutions were prepared by dissolving the necessary quantities of the compound in a Hoagland solution and then the solution pH was adjusted to pH 7.0 by using acid and base. The control plants were grown in a diluted Hoagland nutrient solution with the absence of BPA.

### 2.3 Plant culture and BPA treatment

Korean native soybeans (Glycine max(L) Mrr.) were acquired from the farm for the experiment and kept cold in the fridge at 5 °C until needed. Experiments were performed according to the protocol of Qiu and Wang (2012) with some modifications. Beans were rinsed with distilled water, spread out on cotton gauze, and then incubated in a glass dish at 23 °C to 25 °C for three days. More than 95% of the seeds in the experimental group germinated, and from that population, roughly 100 radicle roots with uniform growth rates were chosen and transplanted to a liquid medium for further study (Hoagland and Arnon nutrient solution). The soybean plants were transplanted in solutions containing varying concentrations of BPA (0, 1, 5, 7, 12, 17.2, 50 mg L^−1^). The control plants were grown in a diluted Hoagland nutrient solution (pH 7). Water loss due to evaporation or plant transpiration was replenished with nutrient solution at weekly intervals. Each control group was cultured in 4 culture tubes and cultured for 28 days at room temperature (23–26 °C) with 9 hours of sunlight to enable continuous growth, the first collection after 14 days and the second collection after 28 days. The growth data results of a total of 12 experimental groups were obtained. This process was repeated four times.

### 2.4 Analysis of growth results

Fresh roots were washed with distilled water to remove moisture with a paper towel, and then the longest length of the root was measured using a tape measure, and the weight was measured using a precision balance. The data measurement of the stem was also made in the same way. True leaves were quantified by the number and weight of leaves. In order to obtain the result value excluding variables of the experimental group growth environment, the minimum value and maximum value were excluded, and the data were confirmed as a total of 8 experimental groups. In order to completely remove the moisture value, it was dried overnight in an oven at 80 °C and the dry weight was remeasured.

### 2.5 Characterization

Field emission scanning electron microscopy (FE-SEM) was performed on the samples using a Hitachi S-4800 II SEM (Japan) instrument. Additionally, the particle size distribution of the samples was analyzed using a Scatter scope I instrument from K-one Nano Ltd. Fourier-transform infrared spectroscopy (ATR-FTIR, Thermo scientific USA) was employed to analyze the functional groups in the samples. In order to examine the samples of roots, stems, and leaves that were exposed to BPA, the GCMS method was employed using a Shimadzu Q2010 GC/MS instrument equipped with an electron capture detector. A column with a length of 60 meters, a diameter of 250 μm, and a thickness of 250 μm (Elite 5 ms column) was utilized. The MS was operated in the EI + mode with an electron energy of 70 eV, and the selected ion monitoring was performed to detect the molecular weight of BPA at *m*/*z* 213. The source temperature was maintained at 250 °C, while the GC transfer line was held at 280 °C. The temperature of the GC oven was programmed to start at 50 °C, gradually increase to 280 °C at 10 °C per minute, further increase to 250 °C at 8 °C per minute, and then maintain at 250 °C. Helium gas was used as the carrier gas at a flow rate of 1 μL per minute. The samples and standards were automatically injected into the GC inlet with a split ratio of 20 : 1 and an inlet temperature of 100 °C. The samples and standards were each injected three times.

## 3. Results and discussions

### 3.1 Characterization of ball-milled BPA particles

Bisphenol A's morphology, particle size, and functional groups were carefully investigated using Field emission scanning electron microscopy (FE-SEM), Dynamic light scattering (DLS), and Fourier-transform infrared spectroscopy (ATR-FTIR), as shown in Fig. 2. Results from scanning electron microscopy (Fig. 2a and b) reveal a particle-like morphology with a rather constant average particle size of around 220 nm. In Fig. 2c, we see the particle size distribution of a BPA solution that has been ball-milled; the findings demonstrate that the BPA particles have an average size of 212 nm. The acquired data show a high degree of correlation with the morphological outcome. In addition, Fig. 2d displays the results of an ATR-FTIR spectroscopic analysis of BPA's functional groups. The purity of BPA is confirmed by FTIR spectra, which exhibit typical peaks at the correct wavenumbers for O–H, aromatic and aliphatic C–H stretching, C–H bending, C–O stretching, and 1,4 aromatic ring bonding.^35,36^

### 3.2 Effects of BPA on soybean biological growth

**Fig. 1:**
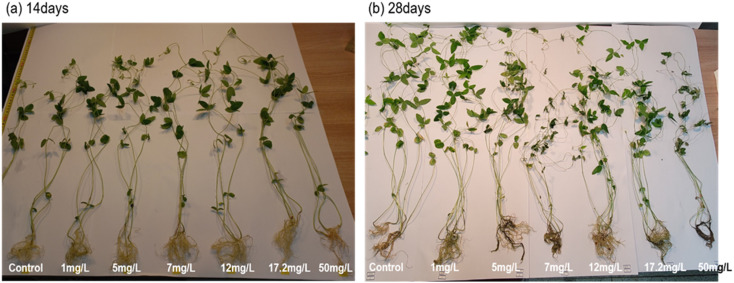
Photographs of soybean plants that have been exposed to different concentrations of BPA (0–50 mg L^−1^) (a) 14 days grown plants and (b) 28 days grown plants.

To completely understand the effect of BPA on the biological growth of soybean plants (14 and 28 days old seedlings), the height and weight of the plant's root, stem and leaves were systematically analyzed and shown in Fig. 3. In terms of root length, a BPA concentration that falls between the range of 1 to 17.2 mg L^−1^ has the ability to encourage the elongation of the tap root in both seedling conditions, but a BPA concentration of 50 mg L^−1^ or more shortens the root length. Similarly, the dry root weight increases with increasing the BPA dosage from 1 to 17.2 mg L^−1^, whereas, 50 mg L^−1^ BPA-treated plant root weight drastically decreased due to higher accumulation of BPA which may damage the growth of plants. It can be clearly seen in Fig. 1. The plant nature is treated by high concertation BPA. In addition, the data set of Fig. 3 is shown Table 1.

**Fig. 2:**
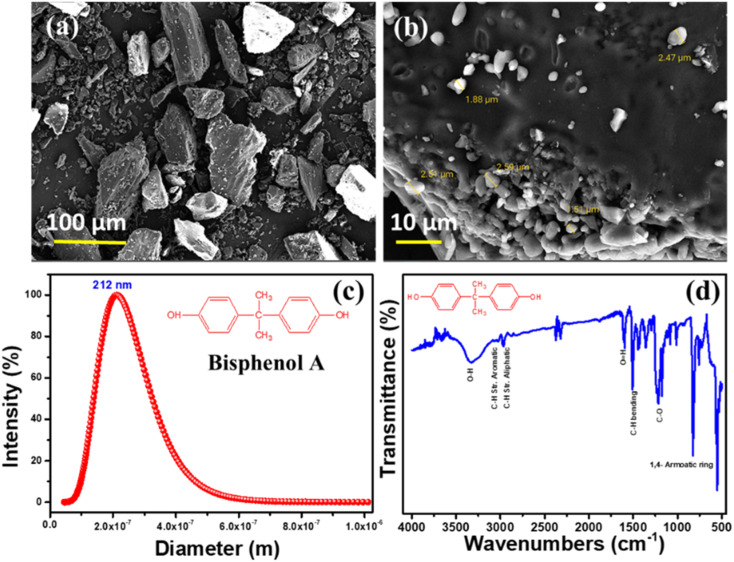
FE-SEM morphological mages (a and b), (c) DLS spectra, and (d) FTIR spectra of ball-milled BPA samples.

**Fig. 3:**
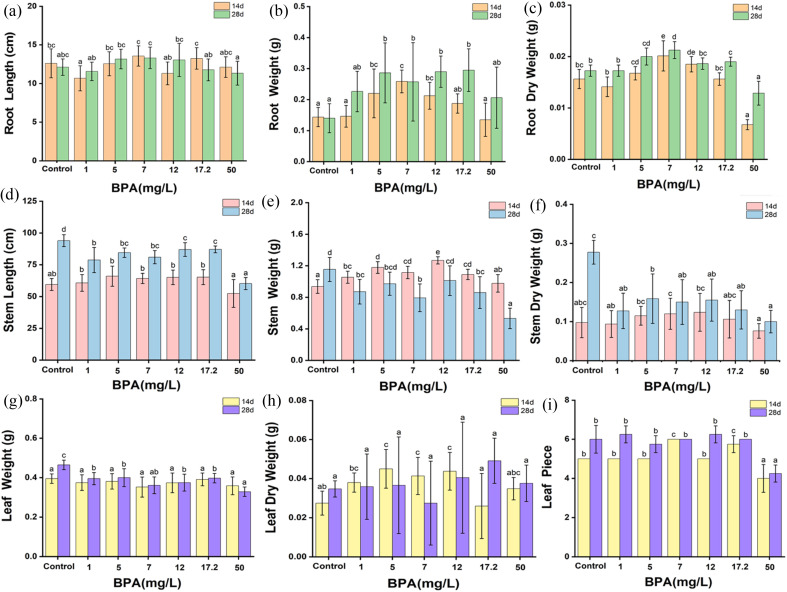
The changes in biological characteristics of (a–c) Soybean roots, (d–f) Stem, and (g–i) leaves after exposure to BPA for 14 days and 28 days. Data are expressed as mean ± SD of four replicates. Bars marked with different letters indicate significant differences (*p* < 0.05).

**Table 1:** Effect of BPAs on the growth of soybeans (Glycine max(L) Mrr.)^a^

Day	BPA (mg L^−1^)	Root			Stem			Leaf		
		Length (cm)	Fresh Weight (g)	Dry weight (g)	Length (cm)	Fresh Weight (g)	Dry weight (g)	Fresh Weight (g)	Dry weight (g)	Number
14	0	12.63 ± 1.87^bc^	0.14 ± 0.03^a^	0.016 ± 0.002^bc^	59.50 ± 4.68^ab^	0.94 ± 0.08^a^	0.098 ± 0.039^abc^	0.40 ± 0.02^a^	0.028 ± 0.006^ab^	5 ± 0^b^
	1	10.69 ± 1.62^a^	0.15 ± 0.03^a^	0.014 ± 0.002^b^	60.75 ± 6.59^b^	1.06 ± 0.07^bc^	0.094 ± 0.034^ab^	0.38 ± 0.04^a^	0.038 ± 0.005^bc^	5 ± 0^b^
	5	12.56 ± 1.55^bc^	0.22 ± 0.08^bc^	0.017 ± 0.001^cd^	66.06 ± 7.91^b^	1.18 ± 0.07^d^	0.115 ± 0.024^bc^	0.38 ± 0.04^a^	0.045 ± 0.010^c^	5 ± 0^b^
	7	13.56 ± 1.31^c^	0.26 ± 0.04^c^	0.020 ± 0.002^e^	64.31 ± 4.02^b^	1.12 ± 0.08^cd^	0.120 ± 0.040^c^	0.35 ± 0.05^a^	0.041 ± 0.009^c^	6 ± 0^c^
	12	11.31 ± 1.48^ab^	0.21 ± 0.04^bc^	0.019 ± 0.002^de^	65.13 ± 5.73^b^	1.27 ± 0.05^e^	0.124 ± 0.048^bc^	0.37 ± 0.05^a^	0.044 ± 0.010^c^	5 ± 0^b^
	17.2	13.25 ± 1.40^c^	0.19 ± 0.03^ab^	0.016 ± 0.001^bc^	65.25 ± 5.86^b^	1.09 ± 0.06^cd^	0.106 ± 0.048^abc^	0.39 ± 0.03^a^	0.026 ± 0.017^a^	5.75 ± 0.43^c^
	50	12.13 ± 1.34^abc^	0.13 ± 0.05^a^	0.007 ± 0.001^a^	52.50 ± 10.95^a^	0.98 ± 0.11^ab^	0.076 ± 0.019^a^	0.36 ± 0.04^a^	0.035 ± 0.006^abc^	4 ± 0.71^a^
28	0	12.13 ± 1.06^abc^	0.14 ± 0.05^a^	0.017 ± 0.001^b^	94.00 ± 4.69^d^	1.15 ± 0.15^d^	0.278 ± 0.030^c^	0.47 ± 0.02^c^	0.035 ± 0.004^a^	6 ± 0.71^b^
	1	11.58 ± 1.20 ^ab^	0.22 ± 0.06 ^ab^	0.017 ± 0.001^b^	78.75 ± 9.92^b^	0.87 ± 0.15^bc^	0.128 ± 0.045 ^ab^	0.40 ± 0.03^b^	0.036 ± 0.017^a^	6.25 ± 0.43^b^
	5	13.18 ± 1.27^bc^	0.29 ± 0.09^b^	0.02 ± 0.002^cd^	84.56 ± 3.70^bc^	0.97 ± 0.15^bcd^	0.159 ± 0.063^b^	0.40 ± 0.05^b^	0.037 ± 0.025^a^	5.75 ± 0.43^b^
	7	13.33 ± 1.40^c^	0.26 ± 0.12^b^	0.021 ± 0.002^d^	81.00 ± 5.22^bc^	0.79 ± 0.17^b^	0.150 ± 0.057^ab^	0.36 ± 0.04^ab^	0.028 ± 0.021^a^	6 ± 0^b^
	12	13.06 ± 2.14^bc^	0.29 ± 0.05^b^	0.019 ± 0.001^bc^	87.00 ± 5.41^c^	1.01 ± 0.18^cd^	0.155 ± 0.054^ab^	0.38 ± 0.04^b^	0.041 ± 0.028^a^	6.25 ± 0.43^b^
	17.2	11.78 ± 1.40^ab^	0.30 ± 0.07^b^	0.019 ± 0.001^c^	87.13 ± 2.71^c^	0.86 ± 0.20^bc^	0.130 ± 0.049^ab^	0.40 ± 0.02^b^	0.049 ± 0.012^a^	6 ± 0^b^
	50	11.34 ± 1.54^a^	0.20 ± 0.10^ab^	0.013 ± 0.002^a^	60.25 ± 4.73^a^	0.53 ± 0.13^a^	0.100 ± 0.029^a^	0.33 ± 0.02^a^	0.038 ± 0.009^a^	4.25 ± 0.43^a^

a The letter a–e shows a significant difference between treatments with different BPAs concentrations at the same particle type and size (*p* <0.05).

Further, the stem length and stem weight of the BPA-treated soybean plant was analyzed and shown in Fig. 3(d–f). Stem length was increased with BPA concentration from 1 to 17.2 mg L^−1^ in both seedling conditions. On the other hand, high-concentration BPA reduces the stem length. In terms of stem weight, 14 day seeded plants have no noticeable changes in the stem weight, in contrast, 28 day seeded plants show drastically decreased in weight with respect to BPA dosage, which clearly confirms that BPA has a significant effect on the plant's growth. In addition, leaf weight and leaf counts were monitored and presented in Fig. 3(g–h). The dry leaf weight linearly increased with respect to BPA dosage, whereas the leaf count was similar up to 17.2 mg L^−1^ BPA, after that the growth of leaves reduced than the control samples.

These results indicated that, in both seedling environments, low-dose BPA promoted the elongation of the tap root, stem length, and leaf counts. In contrast, high-dose BPA inhibited the plant's growth. In summary, BPA exposure adversely affects the biological growth of plants.

### 3.3 Morphological changes in soybean root tissue on BPA exposure

**Fig. 4:**
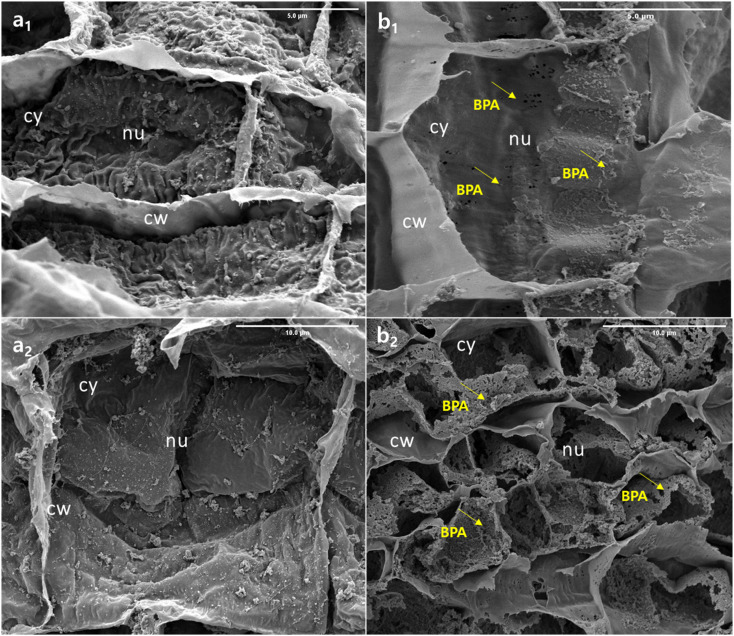
Ultrastructural changes in the root tissue of soybean seedlings exposed to 50 mg L^−1^ BPA for 28 days. (a_1_ and a_2_) Control, and (b_1_ and b_2_) are BPA-exposed samples. Cell wall (cw), cytoplasm (cy), and nucleus (nu).

BPA-exposed (50 mg L^−1^) root tissue was investigated using FE-SEM to track changes in root internal tissue, the findings are shown in Fig. 4. The control sample, designated as (0 mg L^−1^) morphological pictures (a_1_ and a_2_), appears to have maintained a normal ultrastructure thin layer with no visible damage. This is likely due to the fact that these samples were not exposed to BPA, which is the substance that is believed to cause the damage and micropore formation seen in the other tissue samples (b_1_). The tissue samples (b_2_) exposed to BPA show clear signs of damage, as evidenced by the formation of micropores on the tissue surface. This confirms that BPA nanoparticles were able to penetrate through the plant roots. In addition, the detailed morphological features of the root samples are presented in Fig. SI1.† The presence of BPA in the tissue samples suggests that the growth rate, physiological, and biological properties of the soybean plant were negatively impacted by the exposure to BPA. This could lead to decreased productivity and overall health of the plant. Further research is needed to understand the full extent of the effects of BPA on plant growth and health, as well as to develop methods to mitigate or eliminate these negative effects.^37^ The aforesaid, biological changes results are well correlated with the morphological result.

### 3.4 Spectral analysis of BPA penetration in soybean plant

**Fig. 5:**
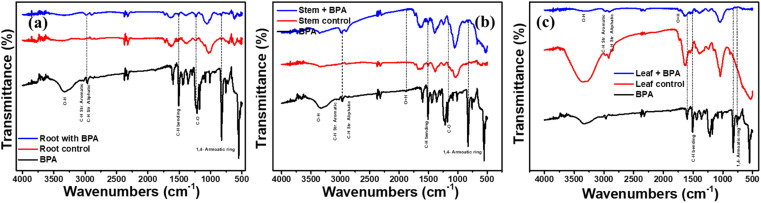
FTIR spectra of (a) root (b) stem and (c) leaf of soybean seedlings exposed to 50 mg L^−1^ BPA for 28 days.

To evaluate the distribution and accumulation of BPA in roots, stems, and leaves were analyzed by using ATR-FTIR spectroscopy. For this analysis, BPA-exposed roots, stems, and leaves were dried and grained separately, and then the fine powder was analyzed in the range of 4000–500 cm^−1^, and the resulting spectra are shown in Fig. 5. It is important to note that, all three samples exhibit BPA characteristic peaks at respective wavenumbers, which confirming the penetration of BPA. BPA-exposed samples (Root, Stem, Leaf) spectra exhibited some new additional peaks than controlled sample spectra. A peak at 3400–3000 cm^−1^, which corresponds to the stretching vibrations of the hydroxyl (O–H) group. The peak at around 1632 cm^−1^ is associated with the bending vibration of the hydroxyl group. A prominent peak is observed in the region of 1470–440 cm^−1^, which corresponds to the stretching vibration of the C–O bond in the ester group. In addition, the peaks in the region of 1150–1100 cm^−1^, corresponding to the bending vibrations of the C–O–C and C–O bonds. The peaks at around 805 cm^−1^ and 723 cm^−1^ correspond to the bending vibrations of the C–H bond in the aromatic ring. Furthermore, BPA peak intensity was higher in the root sample than in the stem and leaf samples, demonstrating that BPA permeated through roots to the stem and leaves of the soybean plant. In addition, the detailed FTIR spectra of BPA exposed root, stem and leaves samples are shown in Fig. SI2.†

In addition, the Gas chromatography-mass spectrometry (GC-MS) method was utilized to examine the samples of roots, stems, and leaves that were exposed to BPA. The spectra obtained from the analysis are displayed in Fig. SI3.† It can be seen that, mass spectra showed the presence of BPA with a characteristic peak appearing at a specific retention time (RT) of 26.9 minutes for all three samples. In addition, the standard BPA solution was analysed by using GC-MS technique to understand the effect of interference species in the BPA exposed plants samples, obtained mass spectra is shown in Fig. SI4.† The mass spectra of standard BPA sample clearly show the intense peak at RT 26.9 minutes with abundant fragment mass *m*/*z* 213 [C_14_H_13_O_2_]^+^. Further, mass spectra fragment of standard BPA is presented in Table SI1.† This result is consistent with the findings from the FTIR analysis, further confirming the presence of BPA in the samples.

## 4. Conclusions

This study provided evidence of BPA was able to penetrate soybean plants and had an influence on the biological growth of the plants. According to the findings of our experiment, the impact of BPA on seed germination and early-stage plant development is depending on the amount of the chemical that is administered. In addition, BPA has the potential to either inhibit or promote the germination of seeds. BPA, even at low levels, prompted an increase in the plant's tap root, stem length, and a total number of leaves. On the other hand, exposure to BPA in high concentrations had the reverse effect and stifled the plant's growth. In general, the findings of these investigations indicate that being exposed to BPA has a negative impact on the growth of plants. The morphological findings revealed that the root tissue had been significantly injured as a consequence of BPA penetration, which ultimately led to the growth of the plant being suppressed. FTIR and GC-MS analysis confirmed the accumulation and distribution of BPA through root to stem and leaves and causes the inhibition of plant growth. In addition, the uptake of BPA by plants can cause adverse effects on food safety through the ecological food chain.

## Conflicts of interest

The authors declare that they have no conflict of interest.

## Author contributions

Eujung Kim: conceptualization, investigation, writing – original draft; Minjung Song: instruments sources, investigation, data curation, visualization; Adam Gopal Ramu: instruments sources, visualization; Dongjin Choi: conceptualization, supervision, writing – original draft, revision, project administration, funding acquisition.

## Supplementary Material

RA-013-D2RA08090G-s001
