# Supplementary material for: Analysis of impacts of exogenous pollutant bisphenol-A penetration on soybeans roots and their biological growth
Source: RSC Adv. 2023 Mar 28;13(15):9781–7. doi: 10.1039/d2ra08090g (PMC10043879; doi:10.1039/d2ra08090g)
Supplement: RA-013-D2RA08090G-s001 [file RA-013-D2RA08090G-s001.pdf]

Electronic Supplementary Material (ESI) for RSC Advances.
This journal is © The Royal Society of Chemistry 2023

Analysis of impacts of exogenous pollutant bisphenol-A penetration on soybeans roots and its biological growth

Eujung Kim [a], Minjung Song [a], Adam Gopal Ramu [a], Dongjin Choi [a]*

[a] *Department of Materials Science and Engineering, Hongik University, 2639-Sejong-ro, Jochiwon-eup, Sejong-city, 30016, Republic of Korea.*

***Corresponding author Email address:** djchoi@hongik.ac.kr (Dongjin Choi)

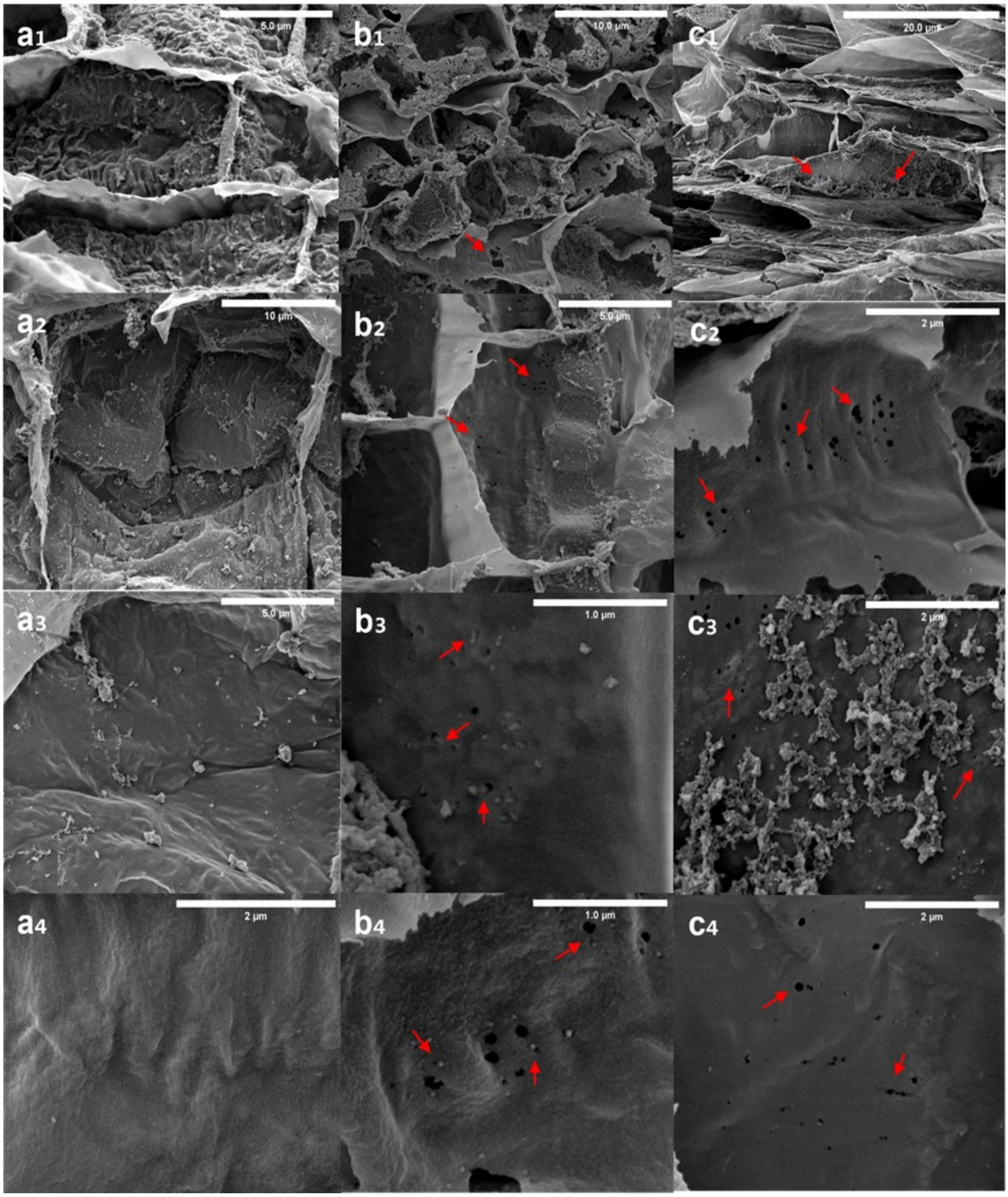

Fig. SI. 1. Ultrastructural changes in the root tissue of soybean seedlings exposed to 50 mg/L BPA for 28 days. (a_1-a_4) Control, (b_1-b_4), and (c_1-c_4) are BPA exposed samples.

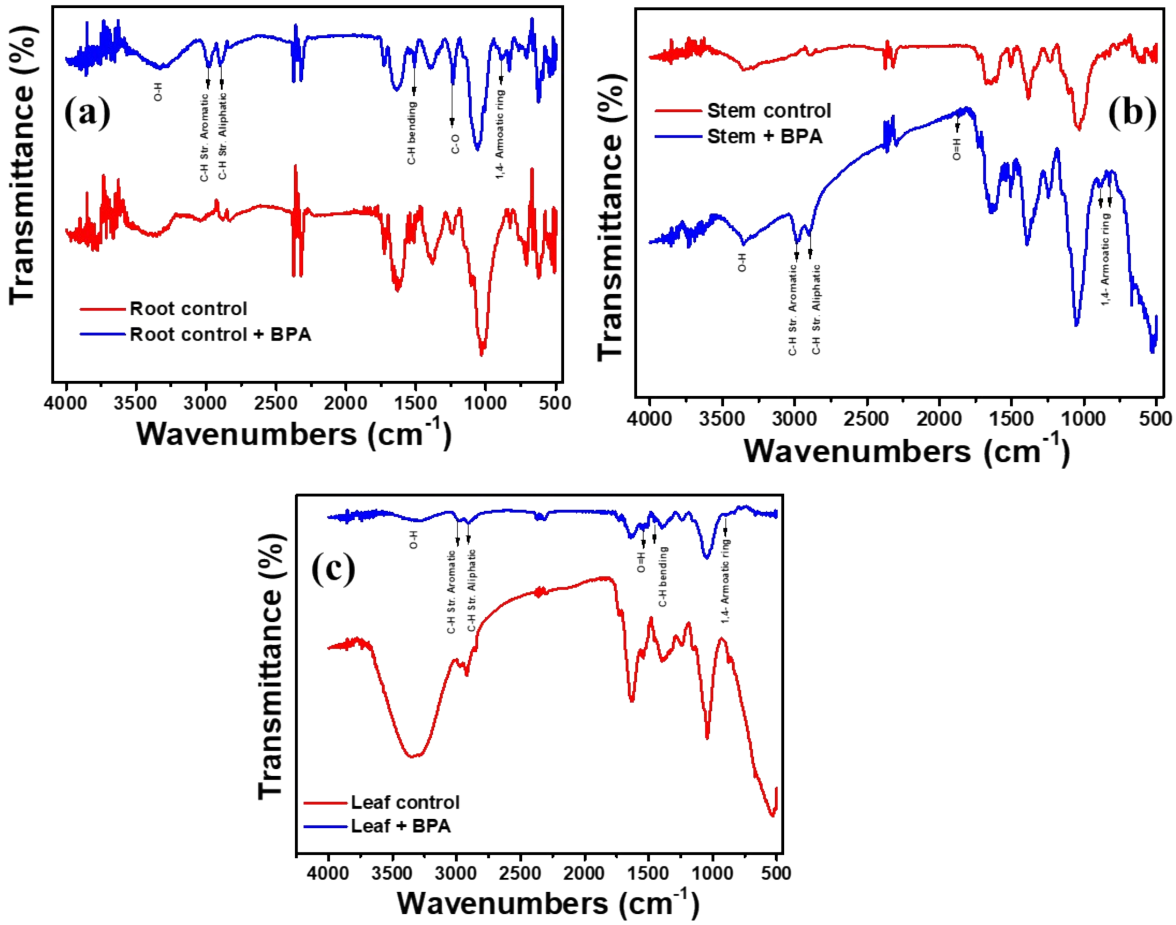

Fig. SI. 2. Detailed FTIR spectra comparison of (a) root (b) stem and (c) leaf of soybean seedlings exposed to 50 mg/L BPA for 28 days.

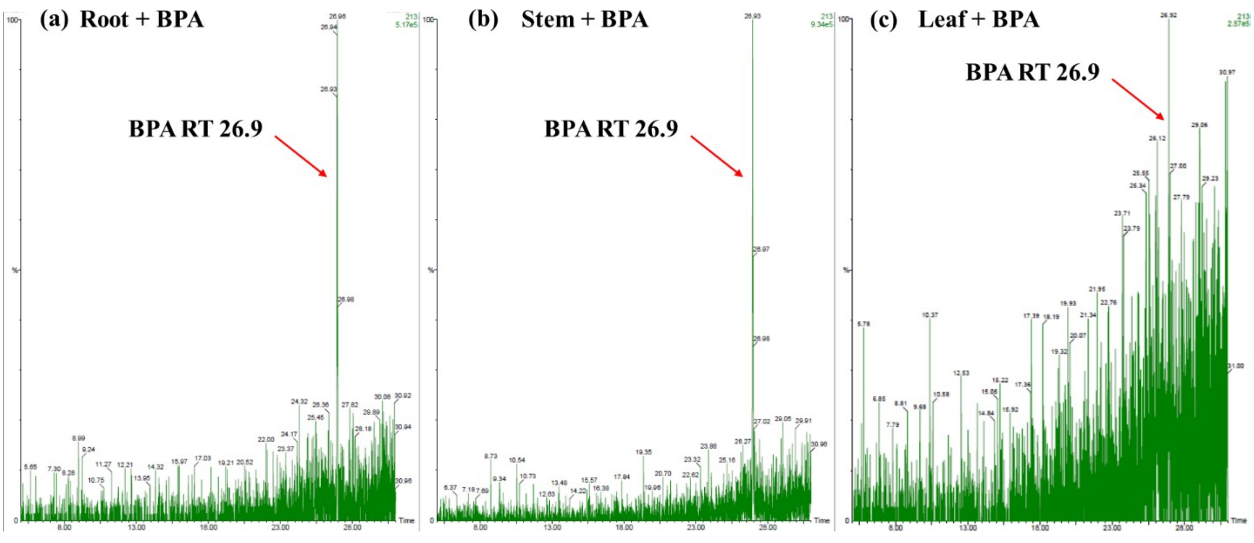

Fig. SI. 3. GC-MS spectra of (a) root (b) stem and (c) leaf of soybean seedlings exposed to 50 mg/L BPA for 28 days.

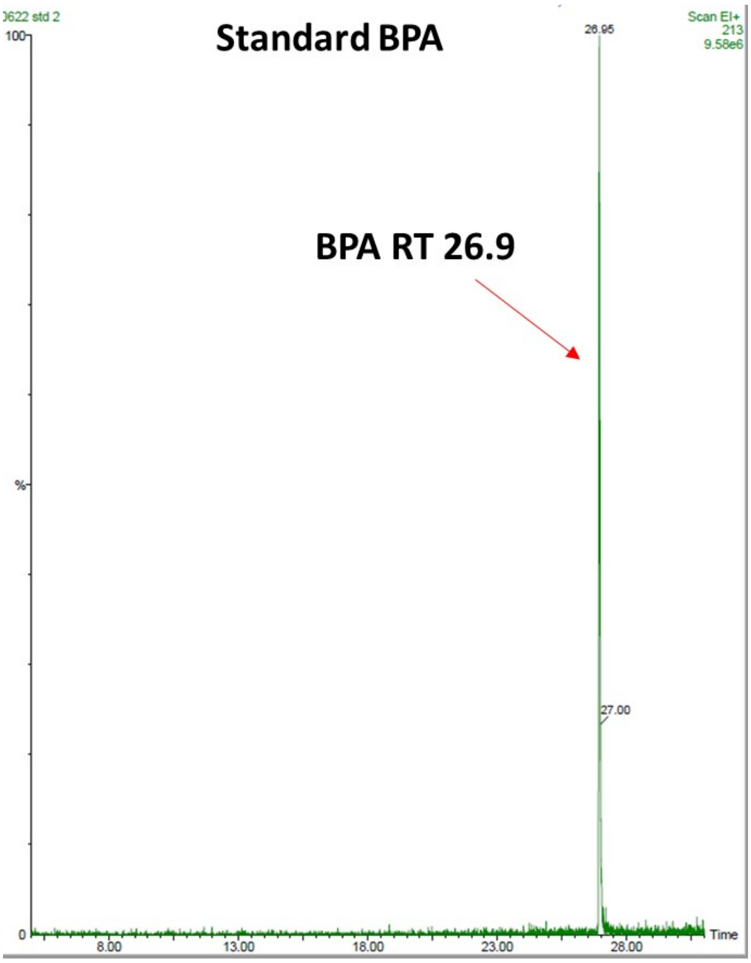

Fig. SI. 4. GC-MS spectra of standard BPA solution.

Table. SI. 1. Mass spectra fragments of standard BPA solution.

**Analyte**	**Precursor ion (m/z)**	**Confirmation**	
		**Product ion (m/z)** $[C_{14}H_{13}O_2]^+$	**Collision energy (eV)**
**BPA**	**228.29**	**213**	**70**
